# Anti-signal Recognition Particle Necrotizing Autoimmune Myopathy: An Atypical Presentation

**DOI:** 10.7759/cureus.3766

**Published:** 2018-12-21

**Authors:** Muhammad H Khan, Abdurraoof Patel, Sima Pendharkar

**Affiliations:** 1 Internal Medicine, The Brooklyn Hospital Center, Brooklyn, USA

**Keywords:** nam, necrotizing autoimmune myositis, srp, signal recognition particle, hmgcr, 3-hydroxy-3- methylglutaryl-coenzyme a reductase, ivig

## Abstract

Necrotizing autoimmune myopathy (NAM), also known as necrotizing autoimmune myositis, is a heterogeneous group of diseases characterized by the presence of necrotic muscle fibers on biopsy, elevated creatine kinase (CK) levels, an abnormal electromyogram (EMG) result, and the associated antibodies. The anti-signal recognition particle (anti-SRP) and the anti-3-hydroxyl-3-methylglutaryl-coenzyme A reductase (anti-HMGCR) antibodies are the two most prevalent antibodies identified with NAM. NAM is a rare disease that typically affects middle-aged Caucasian women. In this case report, we present the diagnosis of anti-SRP NAM in a middle-aged African American male. This case report displays the atypical presentation of NAM outside of the typical patient population as well as the need for additional research to determine the pathogenesis and the precise role of anti-SRP antibodies in NAM.

## Introduction

Idiopathic inflammatory myopathies are a small group of acquired myopathies that are associated with muscle weakness, increased serum creatine kinase (CK) levels, and myopathic findings on electroencephalogram [1-2]. Necrotizing autoimmune myopathy (NAM) represents a heterogeneous group of diseases that are characterized by the presence of necrotic fibers on muscle biopsy along with elevated serum CK levels and an abnormal electromyogram (EMG) result [1-8]. Anti-signal recognition particle (anti-SRP) and anti-3-hydroxy-3-methylglutaryl-coenzyme A reductase (anti-HMGCR) antibodies are the two most common antibodies associated with NAM [1-2]. The clinical features associated with anti-SRP antibody-positive necrotizing myositis most commonly include the rapid progression of proximal muscle weakness, lower-extremity weakness, myalgia, dysphagia, dyspnea, persistently elevated CK, resistance to glucocorticoid and overall, a poor prognostic factor [1-8]. In the United States, there are approximately one in 625,000 cases of NAM per year [1].

## Case presentation

A 58-year-old African American male with autoimmune myositis diagnosed within the past year, and required tracheostomy and percutaneous endoscopic gastrostomy (PEG), was brought to the hospital by paramedics, from a nursing home, due to difficulty in breathing. He had minimal speech capabilities due to the tracheostomy in November 2017 and was subsequently placed on a ventilator. The patient was accompanied by his brother who stated that the patient was disconnected from the ventilator at the nursing home and developed difficulty in breathing. The brother also endorsed that the patient denied chest pain, dizziness, lightheadedness, headaches, palpitations, nausea, vomiting, vision changes, auditory changes, cough, congestion, back pain, abdominal pain, fevers, chills, diarrhea, constipation, or any international travel. The brother also mentioned that the patient had insulin-independent diabetes mellitus, hypertension, hyperlipidemia, chronic obstructive pulmonary disease, and a myocardial infarction status post-percutaneous coronary intervention. The brother denied any family history of malignancy and stated that the patient had no known allergies to medication or food. The patient used to be a basketball referee and had progressively mild weakness in the lower extremities for over a decade. Prior to admission, the patient was taking lisinopril-hydrochlorothiazide 20 mg/25 mg daily, sotalol 80 mg daily, apixaban 5 mg daily, atorvastatin 80 mg daily, metoprolol 100 mg twice a day, prednisone 20 mg daily, mirtazapine 15 mg, Protonix 40 mg daily, and Lantus and Novolog for diabetes mellitus.

Upon arrival at the emergency department, the patient was connected to the ventilator and had stable vital signs otherwise. Physical exam findings were positive for bilateral lower lobe rhonchi, 1 + pitting edema in the lower extremities, weak neck flexor muscles, and severely weak upper- and lower-extremity proximal and distal muscle groups with atrophy of the quadriceps muscles. His ventilation settings were pressure-regulated volume control (PRVC) with the fraction of inspired oxygen (FIO_2_) being 40% and positive end-expiratory pressure (PEEP) of 5 mmHg. His complete blood count (CBC) showed a white cell count of 13.7 K/cm with a left shift. Serum CK and troponin levels were both elevated to 1509 U/L and 0.69 ng/ml, respectively. C-reactive protein levels were also elevated to 9.35 mg/L. Venous blood gas (VBG) showed pH of 7.51 and pCO_2_ of 46 mmHg. On imaging, chest X-ray was positive for a bilateral patchy infiltrate in the lower lobes.

**Figure 1:**
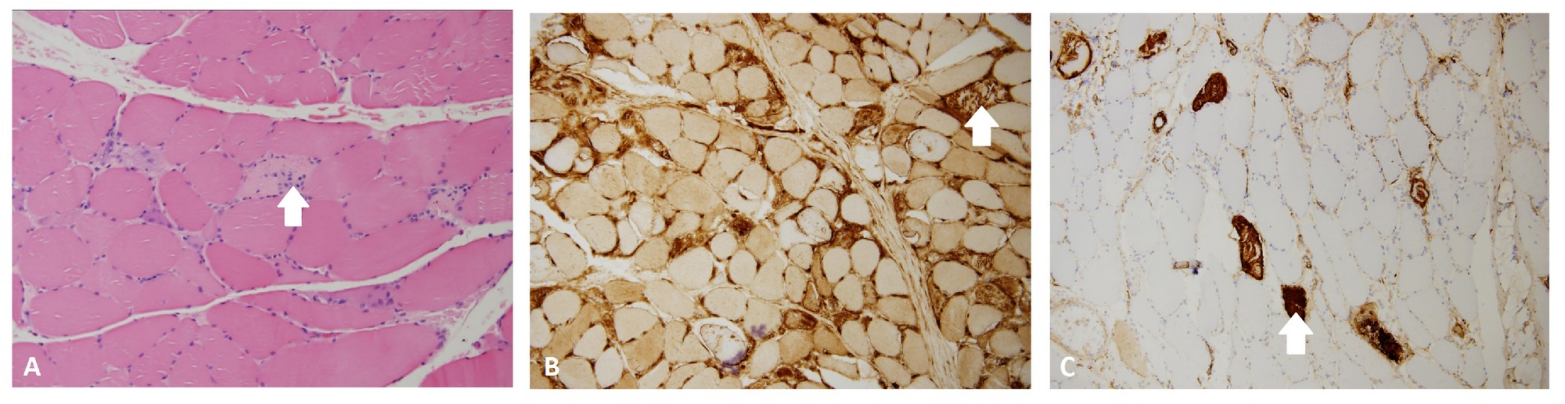
Three types of stained sections of the left deltoid skeletal muscle biopsy showing a necrotizing myopathic process without evidence of significant inflammation A) Hematoxylin- and eosin-stained section; necrotizing myofiber (arrow) B) Major histocompatibility complex class 1 stained section; necrotizing myofiber (arrow) C) Complement 5b-9 stained section; necrotizing myofiber (arrow)

The patient’s medical records were obtained from another institution that showed that the patient had been worked up for NAM, which included autoimmune antibodies and muscle biopsy. As shown in Figure 1, the muscle biopsy revealed “necrotizing myopathic process without any evidence of significant inflammatory process”, a classic pathological finding for NAM. During the current hospital course, an autoimmune panel, including anti-nuclear antibodies (ANA), serum CK, anti-histidyl transfer ribonucleic acid synthetase (anti-Jo-1), anti-ribonucleoprotein (anti-RNP), anti-smooth muscle (anti-SM), anti-3-hydroxy-3-methylglutaryl-coenzyme A reductase (anti-HMGCR), and anti-signal recognition particle (anti-SRP) antibodies, was ordered for further clarification of the serotype of NAM. The patient was solely positive for anti-SRP antibody, with negative ANA and anti-HMGCR antibodies, represented in Table 1.

**Table 1:** Antibodies tested throughout the course of hospital stay

Antibody	
Anti-nuclear antibody (ANA)	NEGATIVE
Anti-smooth muscle (SM)	NEGATIVE
Anti-ribonucleoprotein (RNP)	NEGATIVE
Anti-3-hydroxy-3-methylglutaryl-coenzyme A reductase (HMGCR)	NEGATIVE
Anti-signal recognition article (SRP)	POSITIVE

A computed tomography (CT) scan of the chest and the abdominopelvic region was performed to screen for any possible malignancy leading to NAM. The result of CT scan was negative for any malignancy, ruling out a malignant etiology leading to NAM. In regards to the treatment, the patient received 50 mg of oral prednisone once daily that was tapered down to 5 mg over a five-day course. Due to the refractory nature of the disease, the patient was treated twice with 1000 mg of rituximab given two weeks apart, with each dose supplemented with 100 mg of Solu-Medrol. The patient was discharged and scheduled for a follow-up to monitor the treatment response. 

## Discussion

NAM occurs in about 0.0000016% of the American population annually [1]. Like most autoimmune diseases, NAM is typically seen in middle-aged females [1-3,5-8]. This case emphasizes the outlying population, in terms of the sex and race, that this patient identifies with. Although the etiology of NAM is unknown, some of the risk factors include statin use, malignancy, a human immunodeficiency virus infection, or connective tissue disease [2,4,9]. The patient only had a history of using high doses of Atorvastatin and hence, the diagnosis of NAM was not immediate.

The disease is pathologically defined as having “necrotic muscle fibers with a prominent increase in the endomysial connective tissues and minimal or absence of mononuclear cell inflammation” [2]. This definition signifies that a muscle biopsy is the gold standard of diagnosing NAM [1-8,10]. Immunohistochemical staining also supplements the definition of NAM as the membrane attack complex complement C5b-9 is deposited into capillaries and into the sarcolemma of nonnecrotic myofibers with a broad deposition of major histocompatibility complex class 1 [1-3,5,7-8,10]. The two markers that have commonly been associated with NAM are anti-HMGCR and anti-SRP antibodies [1-10]. These antibodies have been detected in about 66% of the cases of NAM [2]. Historically, patients with anti-HMGCR antibodies tend to have a favorable disease progression as compared to patients with anti-SRP antibodies [1,2,4-7,9]. Supplementary findings are also seen with anti-SRP NAM. With regard to the laboratory values, serum CK levels are typically found to be extremely elevated, numerically in the thousands of units/liter (U/L) [1-8,10]. Additionally, the EMG findings correlated with NAM are described as being "myopathic", which displayed short and low amplitude motor unit potentials [1-2,5,10]. Kassardjian et al. also considered electrocardiography and echocardiography for patients with anti-SRP NAM due to 22% of their cohort having cardiac conduction problems not related to any previous cardiac history [8].

NAM with anti-SRP antibodies is characterized by rapidly progressive proximal muscle weakness, markedly elevated CK levels, and poor responsiveness to corticosteroid therapy as seen in this case [1-2,4-8]. Intravenous immunoglobulin (IVIG) therapy, plasmapheresis, or immunosuppression with methotrexate, azathioprine, rituximab, cyclophosphamide, or mycophenolate mofetil seem to be alternative options [1]. Early administration of therapy of corticosteroids with an immunosuppressant, within three months, was found to be beneficial [2,6]. Although, according to Kassardjian et al., the early initiation of IVIG was seen to be advantageous [8]. Suzuki et al. found that oral corticosteroids and IVIG were adequate as primary treatment and plasmapheresis was useful as the secondary treatment [6]. However, there has not been an official regimen for treating anti-SRP NAM [7-8]. A case series of eight patients demonstrated a beneficial clinical response to the B-cell depletion therapy with rituximab after they had failed on other immunosuppressive therapies [10]. Similarly, this patient received two doses of rituximab, two weeks apart, with the pretreatment with Solu-Medrol in order to obtain the effectiveness of treatment [10]. There seems to be a high rate of relapse when completing or tapering treatment [2,5,8].

## Conclusions

We conclude that this case requires to be monitored longitudinally to observe for the course of symptoms, adverse events, and outcomes as this patient is an outlier to the typical population that is diagnosed with anti-SRP NAM. Further studies are also required to elucidate the pathogenesis and the precise role of anti-SRP antibodies in NAM.
